# To bind or not to bind: Cistromic reprogramming in prostate cancer

**DOI:** 10.3389/fonc.2022.963007

**Published:** 2022-09-23

**Authors:** Michelle Shen, Léa-Kristine Demers, Swneke D. Bailey, David P. Labbé

**Affiliations:** ^1^ Cancer Research Program, Research Institute of the McGill University Health Centre, Montréal, QC, Canada; ^2^ Division of Experimental Surgery, Department of Surgery, McGill University, Montréal, QC, Canada; ^3^ Division of Experimental Medicine, Department of Medicine, McGill University, Montréal, QC, Canada; ^4^ Division of Thoracic Surgery, Department of Surgery, McGill University, Montréal, QC, Canada; ^5^ Division of Urology, Department of Surgery, McGill University, Montréal, QC, Canada

**Keywords:** cistromic plasticity, prostate cancer, cistromic reprogramming, transcriptional regulation, epigenetics

## Abstract

The term “cistrome” refers to the genome-wide location of regulatory elements associated with transcription factor binding-sites. The cistrome of key regulatory factors in prostate cancer etiology are substantially reprogrammed and altered during prostatic transformation and disease progression. For instance, the cistrome of the androgen receptor (AR), a ligand-inducible transcription factor central in normal prostate epithelium biology, is directly impacted and substantially reprogrammed during malignant transformation. Accumulating evidence demonstrates that additional transcription factors that are frequently mutated, or aberrantly expressed in prostate cancer, such as the pioneer transcription factors Forkhead Box A1 (FOXA1), the homeobox protein HOXB13, and the GATA binding protein 2 (GATA2), and the ETS-related gene (ERG), and the MYC proto-oncogene, contribute to the reprogramming of the AR cistrome. In addition, recent findings have highlighted key roles for the SWI/SNF complex and the chromatin-modifying helicase CHD1 in remodeling the epigenome and altering the AR cistrome during disease progression. In this review, we will cover the role of cistromic reprogramming in prostate cancer initiation and progression. Specifically, we will discuss the impact of key prostate cancer regulators, as well as the role of epigenetic and chromatin regulators in relation to the AR cistrome and the transformation of normal prostate epithelium. Given the importance of chromatin-transcription factor dynamics in normal cellular differentiation and cancer, an in-depth assessment of the factors involved in producing these altered cistromes is of great relevance and provides insight into new therapeutic strategies for prostate cancer.

## 1 Introduction

Prostate cancer is the second most prevalent cancer in men worldwide with an estimated 1.4 million new cases globally in 2020 (1). In the United States, it is one of the most commonly diagnosed cancers among men (2). Despite established therapeutic strategies such as active surveillance, surgery, or radiation therapy, prostate cancer is still projected to be the second leading cause of cancer-related deaths among men in the United States in 2022 (2).

*Trans*-acting factors, such as transcription factors, regulate the transcription of genes upon binding to their DNA recognition motifs within target *cis-*regulatory elements (3, 4). These *cis*-regulatory elements are flanked by nucleosomes with specific post-translational modifications to their histone proteins. These histone modifications specify different chromatin states and permit or limit the access of these *trans*-acting factors to their target *cis*-acting elements (5). The specific epigenetic modifications and their roles in regulating chromatin states in various cancer contexts have been reviewed elsewhere (6, 7). The term “cistrome” is used to describe the genome-wide locations of transcription factor binding-sites (8). Therefore, the cistrome encompasses the complete set of target *cis-*regulatory elements, including promoters, enhancers, and silencers that are bound or marked by these regulatory factors.

Although inherently dynamic, a tightly regulated epigenome is crucial to maintain cell identity and normal cellular physiology. Disruption of the established chromatin states, such as the inactivation of active regulatory elements and/or the reactivation of inactive regulatory elements, can lead to the establishment of various oncogenic programs (5). Studying these interactions and alterations in the context of prostate cancer raises exciting possibilities. Chromatin remodeling and epigenetic dysregulation leading to cistromic reprogramming — that is, the use of alternative *cis*-regulatory elements — has been increasingly recognized as a hallmark of prostate cancer initiation and progression (8–10). The androgen receptor (AR) signaling axis plays a central role in prostate cancer progression and is the prime target of modern-day prostate cancer phamacopeia (11, 12). Transcription factors and epigenetic modifiers may influence this signaling axis throughout disease progression by way of reprogramming the AR cistrome. In the following review, we will discuss the transcription factors FOXA1, HOXB13, GATA2, ERG, and MYC as they relate to the AR cistrome and its transcriptional output during prostate cancer evolution. Similarly, we will discuss the roles that global chromatin remodelers SWI/SNF and CHD1, as well as the epigenetic regulator EZH2, play in the reprogramming of the AR cistrome. Notably, studying the plasticity of the AR cistrome has revealed several opportunities for therapeutic intervention, and these will be highlighted in the review as well.

## 2 Alterations to the AR cistrome are central to prostate cancer progression

**Figure 1:**
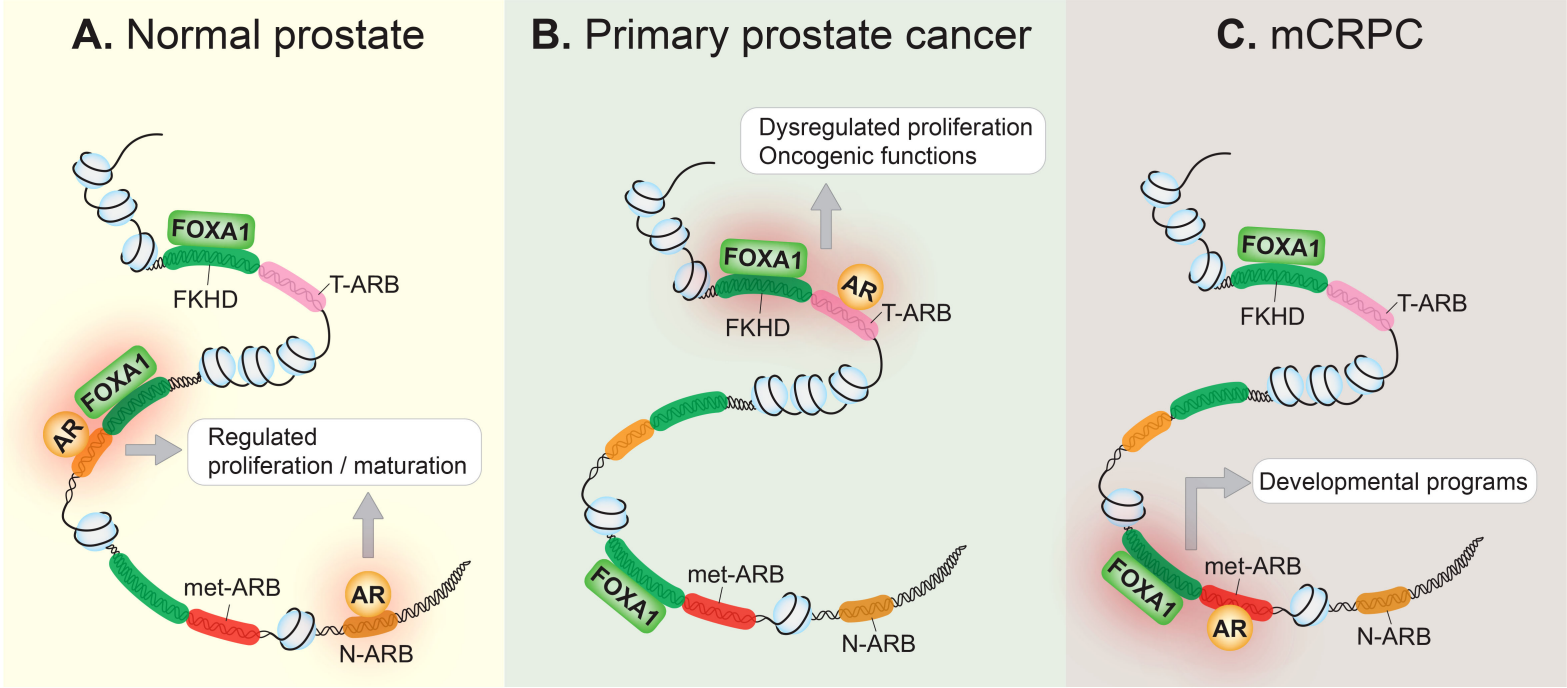
The AR cistrome is reprogrammed during prostate cancer onset and progression. **(A)** In the normal prostate epithelium, the AR occupies its normal cistrome, the collective of normal AR binding sites (N-ARB), and controls the AR signaling axis to regulate cellular proliferation, and the maturation of the prostate gland. Pioneer transcription factors, such as FOXA1 which binds to the forkhead (FKHD) binding motif, contribute to AR-regulated transcription. **(B)** In primary prostate cancer, the AR is redirected to a distinct set of tumor-associated AR binding sites (T-ARB), resulting in aberrant cellular proliferation, transforming the AR into an oncogene. This may be due to genetic or epigenetic disturbances that disrupt the homeostatic relationship between FOXA1 and the AR in normal prostate epithelium. **(C)** In metastatic castration-resistant prostate cancer (mCRPC), the AR cistrome is further reprogrammed, and AR binds to metastatic AR-binding sites (met-ARB), activating decommissioned developmental programs, seemingly to drive metastasis. FOXA1 binding at these metastatic-associated binding sites precedes the development of mCRPC.

AR signaling plays a key role in normal prostate development as well as in prostate cancer pathogenesis (13). Modulation of AR signaling underlies prostate cancer progression and can be partly attributed to the reprogramming of the AR cistrome during prostate tissue transformation. Indeed, several studies have illustrated how the AR cistrome, together with AR-dependent transcriptional networks, evolve throughout clinical disease stages. For example, by using both normal and primary human prostate tissue samples, Pomerantz and colleagues showed that the AR cistrome in primary prostate tumors is distinct from that of normal tissue (14). Additionally, they subsequently showed that the AR cistrome is dramatically and further transformed in metastatic tissues when compared to primary disease (15). Specifically, they found that the metastatic AR cistrome appears to reactivate developmental programs of the prostate. The epigenetic landscape in metastatic castration-resistant prostate cancer (mCRPC) bears similarity to fetal UGS cells derived from the human urogenital sinus, from which the prostate eventually develops (15). In line with this, Wang and Koul found that loss of the canonical AR transcriptome was associated with tumor metastasis and poor clinical outcomes (16). Altogether, this suggests that prostate tumors reactivate decommissioned developmental programs, thereby achieving the traits necessary for metastasis. These changes are summarized in **Figure 1**.

In addition to the changes that occur during progression to metastatic disease, alterations to the AR cistrome also underly the development of therapeutic resistance in prostate cancer patients. With regards to reprogramming of the AR cistrome in response to androgen deprivation therapy (ADT), Han and colleagues observed that the AR cistrome remains largely conserved from hormone-sensitive prostate cancer (HSPC) to CRPC (17). This contrasts the findings of Pomerantz and colleagues, where they observe a clear distinction between the AR cistromes of primary prostate cancer and mCRPC patient samples (15). This discrepancy may be explained by the types of models used in these studies. Han and colleagues used a xenograft model of castration-resistance derived from the hormone-sensitive VCaP cell line (17). On the other hand, Pomerantz and colleagues examined the AR cistromes of human mCRPC biopsies and patient-derived xenografts (PDXs) obtained from human mCRPC samples (15). Thus, the alterations to the AR cistrome observed by Pomerantz and colleagues in mCRPC, such as those associated with prostate developmental programs, may be more relevant for prostate cancer metastasis, rather than the development of castration-resistance *per se*. However, reprogramming of the AR cistrome is observed during the development of resistance to second-generation AR signaling inhibitors (ARSI), such as enzalutamide or abiraterone acetate (18, 19). While the AR signaling axis is largely conserved in CRPC — mainly due to various adaptive mechanisms within the cancer cell that preserve the AR transcriptional program despite low availability of androgens — resistance to ARSI is often marked by loss of AR and independence from its signaling axis (13, 20). This manifests phenotypically as CRPC with neuroendocrine features (CRPC-NE), although other subtypes such as the double-negative (negative for both AR and neuroendocrine markers) subtype or a stem cell-like subtype have been documented (13, 21). Neuroendocrine prostate cancer (NEPC) occurs very rarely *de novo*, accounting for fewer than 2% of all prostate cancers, whereas treatment-induced NEPC occurs in an estimated 10-17% of CRPC patients (22). Mechanisms of therapeutic resistance in relation to the AR signaling axis — including the gain of AR mutations and the expression of AR splice variants during the transition from HSPC to CRPC, along with the upregulation of novel lineage-specific transcription factors in response to ARSI-resistance — have been more broadly reviewed elsewhere (13, 20). Finally, in addition to therapies that aim to suppress the AR signaling axis, supraphysiological levels of testosterone (SPT) that activate the AR signaling axis are also an effective treatment option in a subset of patients with CRPC (23). Critically, tumors that respond to SPT exhibited a distinct AR cistrome compared to tumors that do not respond to SPT (23). This suggests that differences in the AR cistrome may underly differential responses to SPT.

Therefore, reprogramming of the epigenome and chromatin landscape are increasingly becoming recognized mechanisms of disease progression and therapeutic resistance. Given the abundant evidence supporting the role of epigenetic and chromatin regulators in prostate cancer progression, we strove to summarize how a few of these key players converge on the AR cistrome and the AR transcriptome in this review. Studying how the AR cistrome is transformed involves examining how the AR interacts with other key transcription factors in prostate cancer that can dictate its chromatin binding activity, as well as examining widespread chromatin alterations which can alter the AR cistrome more globally. Other mechanisms of altering the AR cistrome, including AR mutations, the expression of splice variants, are beyond the scope of this review.

## 3 Key transcription factors in prostate cancer regulate the AR cistrome and its transcriptional network

### 3.1 Pioneer transcription factors influence AR binding events during disease progression

Pioneer transcription factors are a class of transcription factors that possess the unique ability to bind DNA motifs located within condensed regions of chromatin (24). By so doing, they initiate a process of transcriptional activation by recruiting epigenetic modifiers, which increase the accessibility of the region to other transcription factors (24). Thus, they have earned the moniker “pioneer”. Owing to their role in governing and maintaining chromatin states, pioneer transcription factors have key roles in determining cell identity and cell fate (25).

Several pioneer transcription factors are recognized as drivers of prostate cancer initiation and progression, including the forkhead box A1 (FOXA1) transcription factor, the homeobox protein HOXB13, and the GATA binding protein 2 (GATA2). Their functional characterization, mutations, aberrant expression patterns, and potential for therapeutic targeting in prostate cancer settings have been more exhaustively reviewed elsewhere (24). Here, we provide a review of the relevance of these factors in prostate cancer progression with respect to how they modify the AR cistrome.

#### 3.1.1 FOXA1 and the AR

FOXA1 is a direct binding partner of and an extensively characterized pioneer factor for the AR (24, 26, 27). Indeed, FOXA1 is crucial to normal prostate development, where it cooperates with the AR to drive the growth and survival of normal prostate cells (**Figure 1A**) (28). A landmark study showed that FOXA1 plays a crucial role in opening genomic regions and cooperating with the AR at cell type-specific enhancers to regulate the expression of androgen-stimulated genes (27). Studies using a variety of prostate cancer cell lines later demonstrated that FOXA1 overexpression or knock-down reprograms the AR cistrome (29–32). While FOXA1 is not necessary for AR chromatin binding, it can redirect the AR to bind at sites harboring the forkhead (FKHD) motif (31, 32). Moreover, transduction of FOXA1 in an immortalized prostate epithelial cell line redirected the AR from binding at normal tissue-associated AR binding sites to instead occupy tumor-associated AR binding sites (14). Altogether, this strongly suggests that FOXA1’s pioneering activity contributes to prostate cancer tumorigenesis by influencing the AR cistrome. A more recent study showed that the reprogrammed AR cistrome observed in mCRPC coincides with sites already bound by FOXA1 in normal and primary tumor tissue (15), further supporting a model where FOXA1 pre-marks a cancer-associated AR cistrome to facilitate prostate cancer progression (**Figures 1B, C**). These findings are in line with the pioneering role of FOXA1, since pioneer transcription factor binding precedes the binding of downstream transcription factors. For example, pioneer transcription factors are known to mark transcriptionally silent but competent genes — in other words, genes that later become transcriptionally active in response to an inductive signal (33). This, in combination with the finding that FOXA1 is a key transcription factor in mCRPC models that rely on the AR signaling axis (21), posits FOXA1 as a crucial contributor to AR-dependent prostate cancer progression. Although observed in clinical samples, whether the metastatic AR cistrome would exist in the absence of the pioneering activity of FOXA1 has yet to be experimentally evaluated.

The *FOXA1* gene is recurrently mutated in prostate tumors and *FOXA1* mutations define a specific molecular subtype in the TCGA cohort (34). These findings have fueled recent interest in interrogating the role of FOXA1 mutations on prostate cancer progression. Interestingly, the frequency of FOXA1 mutations in primary prostate tumors is much higher in a Chinese cohort of prostate cancer patients compared to the largely European-centric cohort of the TCGA, with a frequency of ~41% in the former cohort compared to 4% in the latter (35), suggesting ethnic variation with regards to FOXA1 mutations among prostate cancer patients. FOXA1 is frequently mutated in mCRPC as well (36, 37). In an aggregate cohort of prostate cancer patients largely based in institutions located in Western countries, the frequency of FOXA1 mutations among patients with primary disease was approximately 8-9% while among patients with metastatic disease, the frequency of FOXA1 mutations rose to between 12 and 13% (36). Given the established role of FOXA1 in regulating AR activity in prostate cancer, much interest has been focused on the interplay between FOXA1 mutations and the AR cistrome. However, the effect on the AR cistrome seems to vary depending on the type of FOXA1 mutation studied, and these effects are succinctly summarized in a review by Teng and colleagues (28).

**Figure 2:**
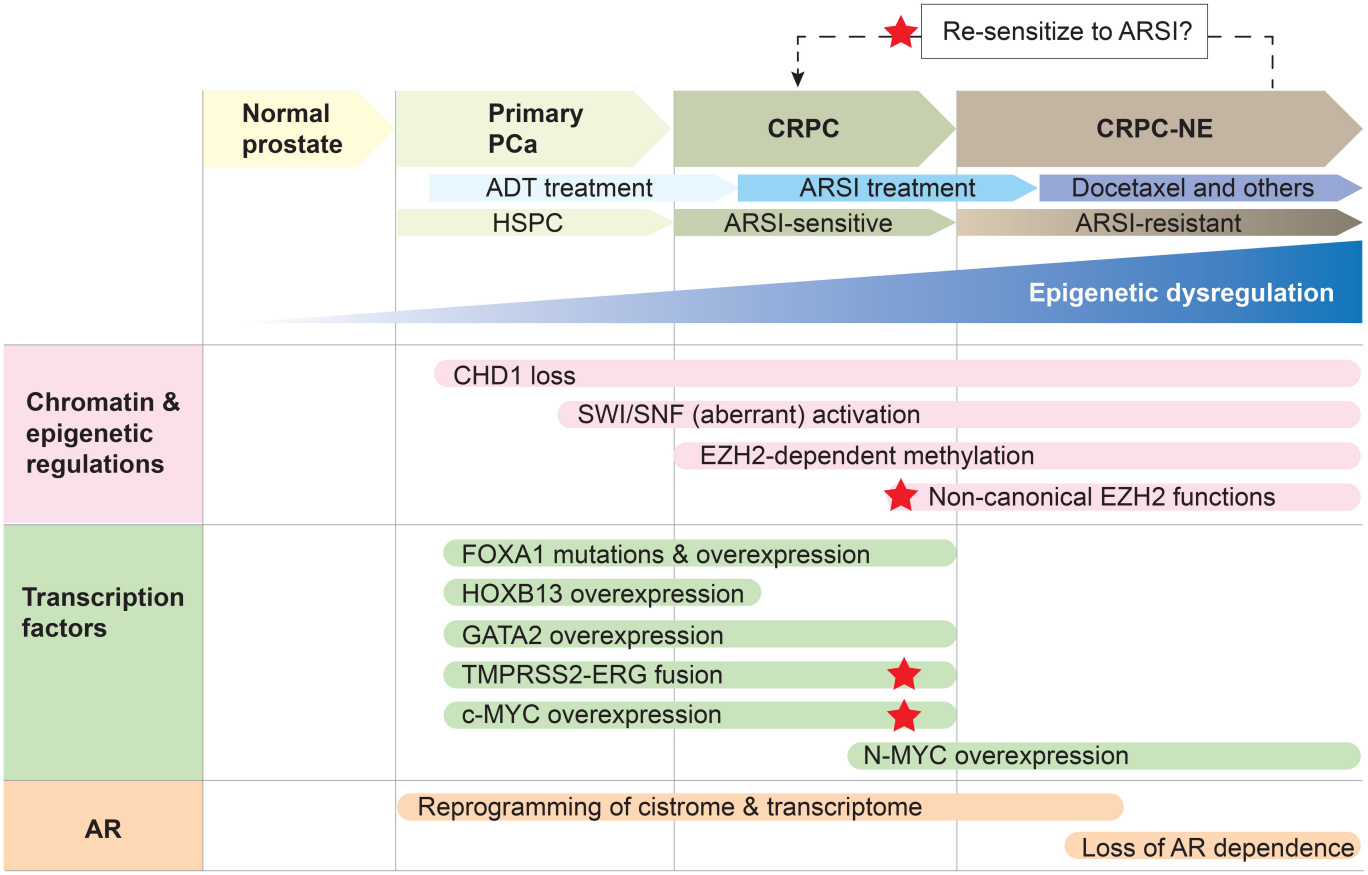
Epigenetic dysregulation and concordant aberrant transcription factor activity result in a reprogrammed AR cistrome and transcriptome to drive prostate cancer progression. Primary prostate cancer (PCa) is hormone-sensitive (HSPC) and responds to androgen-deprivation therapy (ADT). Resistance to ADT results in the development of castration-resistant prostate cancer (CRPC), which remains reliant on AR signaling and is therefore responsive to second-generation AR signaling inhibitors (ARSI; *e.g.*, abiraterone acetate, enzalutamide). However, resistance to ARSI is accompanied by the onset of CRPC with neuroendocrine features (CRPC-NE), which can be treated with chemotherapy such as docetaxel. Reprogramming the cancer cell to an ARSI-sensitive state is an attractive therapeutic strategy, and red stars denote key players that may be targetable to achieve a reversal in treatment-resistance. Note that this is one example of how prostate cancer may be treated, with an emphasis on how the AR signaling axis is therapeutically targeted. Epigenetic dysregulation underlies prostate cancer progression and the development of treatment resistance to ADT and second-generation ARSI. CHD1 loss, accompanied by the overexpression and aberrant activities of the SWI/SNF remodeling complexes, EZH2 and PRC2, result in a plastic epigenome. Epigenomic plasticity also provides cancer cells the opportunity to develop AR-independent mechanisms of tumor growth when the AR signaling pathway is exposed to more extensive inhibition through ARSI treatment. This, in combination with the hyper-activity or altered activity of pioneer transcription factors FOXA1 and HOXB13 as well as the transcription factors ERG, c-MYC and N-Myc, result in enhancer rewiring and reprogramming of the AR cistrome, thereby driving disease progression and unfavorable therapeutic responses. The pioneer transcription factor GATA2 is a key cofactor for maintaining the AR transcriptional program during HSPC and CRPC — however, it does not seem to reprogram the AR cistrome in the same way that FOXA1 and HOXB13 do, suggesting there may be a hierarchy in pioneer transcription factor activity.

Altogether, owing to its role in pioneering, or unpacking the chromatin, for the AR, FOXA1 poses as a key prostate cancer-specific regulator of disease progression and therapeutic response. Although historically a challenging protein to target, recent studies have uncovered post-translational modifications that positively regulate FOXA1 activity, including demethylation by lysine-specific demethylase 1 (LSD1) and methylation by EZH2, thereby positing FOXA1 as a promising target of LSD1 and EZH2 inhibitors (38, 39). Specifically, LSD1 demethylates lysine residue 270, thereby stabilizing FOXA1-chromatin interactions (38). On the other hand, EZH2 methylates lysine residue 295 which leads to de-ubiquitination and enhanced FOXA1 protein stability (39). While the impacts of these pharmacological inhibitors on FOXA1 activity and prostate cancer growth are still limited to the preclinical stage, there are opportunities to test these findings clinically. LSD1 inhibitors are undergoing phase 2 clinical trials for other cancer types, and multiple small-molecular EZH2 inhibitors have been approved and/or are in clinical trials for other cancer types (39, 40). Importantly, therapeutic strategies targeting the link between FOXA1 and the AR would be relevant in disease stages where AR signaling continues to be critical and where FOXA1 expression remains high, such as primary prostate cancer and CRPC (**Figure 2**).

#### 3.1.2 HOXB13 and the AR

HOXB13 is a pioneer transcription factor expressed in normal and cancerous prostate tissue (41). *HOXB13* is rarely mutated among prostate cancer patients. For example, a targeted sequencing study of a cohort of 5,083 prostate cancer patients has shown that 1.4% of prostate cancer patients have a recurrent G84E mutation in *HOXB13*, which increases in prevalence to 3.1% among early-onset prostate cancer patients with family history of the disease (42). However, even when not mutated, *HOXB13* transcript levels are higher in clinical samples of localized disease compared to adjacent normal tissue, suggesting that *HOXB13* overexpression may be a feature of primary prostate cancer (41). On the other hand, *HOXB13* transcripts are downregulated in mCRPC relative to primary prostate cancer, which suggests that its involvement in modifying the AR cistrome may be more relevant to early stages of the disease (43). A more comprehensive review of studies that have investigated the oncogenic potential of HOXB13 in prostate cancer is offered elsewhere (24).

HOXB13, like FOXA1, is a pioneer transcription factor involved in establishing a cancer-associated AR cistrome (14, 15). HOXB13 binding sites overlap extensively with tumor-associated AR binding sites in clinical specimens, and transduction of *HOXB13* in an immortalized normal prostate epithelial cell line is capable of reprogramming the AR cistrome. However, co-transduction of *FOXA1* and *HOXB13* together ultimately results in the most dramatic shift in the AR cistrome away from normal prostate-associated AR binding sites towards tumor-associated AR binding sites (14). Finally, as with FOXA1, HOXB13 seems to occupy ‘sentinel’ sites in prostate tissue where AR is destined to bind later during disease progression (15). Thus, akin to FOXA1, HOXB13 appears to possess important functions in dictating the AR cistrome, primarily in localized disease (**Figure 2**).

Interestingly, HOXB13 seems to have a particular role in governing the cistrome of the AR splice variant 7 (AR-V7), a constitutively active AR splice variant that may confer resistance to AR-targeting therapies (13, 44). While the cistromes of endogenous AR-V7 in two CRPC cell lines grown in hormone-depleted media were distinct from each other, they both overlapped with the HOXB13 cistromes of their respective cell lines (44). HOXB13 is a direct binding partner of AR-V7 and they cooperatively upregulate a subset of genes in both cell lines, although the subset of genes is distinct between the cell lines (44). These results suggest that even though AR-V7 may regulate heterogenous transcriptional programs in different CRPC tumors, the colocalization of HOXB13 at AR-V7 binding sites may be a homogenous and targetable feature (44). How these results fit with the observation that HOXB13 appears downregulated in mCRPC, compared to primary prostate cancer, remains to be determined (43). As described earlier, this discrepancy may be attributed to the differences in cancer biology between mCRPC and non-metastatic CRPC disease and the cancer models used to study them. While some CRPC cell lines may be derived from prostate cancer metastases and may express CRPC-associated markers, such as the AR-V7 splice variant, they are imperfect models for studying metastatic disease.

Altogether, HOXB13 appears to be involved in prostate oncogenesis as well as in the development of therapeutic resistance to androgen-targeting therapies *via* its role in regulating the AR cistrome and the cistrome of its splice variant, AR-V7. However, to better dissect the role of HOXB13 in prostate cancer, both the clinical stage of the disease and the history of AR-targeting therapies should be considered when evaluating clinical data. Similarly, the relevance of the cellular and animal models used to study the role of HOXB13 should also be evaluated against the clinical outcomes that they are meant to represent. By doing so, we may find new ways to reconcile seemingly opposing findings regarding the role of HOXB13 in prostate cancer.

#### 3.1.3 GATA2 and the AR

Finally, GATA2, another pioneer transcription factor, is crucial for the activation of AR signaling in prostate cancer. As a pioneer transcription factor, GATA2 makes regulatory regions accessible to the AR by recruiting the histone acetyltransferase p300 (45). Furthermore, GATA2 binds to AR-target enhancers prior to androgen stimulation and AR DNA-binding, further illustrating its role as a pioneer transcription factor for the AR (45).

Interestingly, previous studies have shown that GATA2 and FOXA1 co-occupy 55% of all hormone-responsive AR binding sites in the LNCaP prostate cancer cell line, suggesting a potential cooperative mechanism between these two pioneer transcription factors in regulating the AR cistrome (45). Indeed, studies have shown that GATA2 can enhance both the AR cistrome and the FOXA1 cistrome by promoting AR and FOXA1 binding at androgen-regulated enhancers (46). However, unlike FOXA1, GATA2 does not seem to reprogram the AR cistrome by directing the AR to bind at a novel set of binding sites. Instead, the primary role of GATA2 appears to be that of an amplifier of AR DNA-binding in hormone-responsive disease stages (**Figure 2**) (46).

GATA2 has been implicated in later-stage, lethal prostate cancers as well. Notably, GATA2 expression is progressively elevated from primary disease to CRPC in clinical specimens (47). In addition, the upregulation of GATA2 is associated with chemoresistance in the prostate cancer cell lines DU145 and 22Rv1, and GATA2 expression is likewise highest in CRPC patients treated with chemotherapy (47). At the CRPC stage where AR signaling is often still central to tumor growth, GATA2 continues to act as a crucial cofactor for AR transcriptional activity and colocalizes with both full-length AR and AR splice variants on the chromatin (48, 49). Thus, as is the case during HSPC, GATA2 contributes to prostate cancer growth during CRPC by amplifying the AR transcriptional program. Yet, GATA2 was found to regulate a set of AR-independent genes in a castration-resistant and chemotherapy-resistant xenograft model of prostate cancer (47). This subset of genes was enriched in men with lethal prostate cancer and patients who had received chemotherapy (47). Therefore, upon advancement to lethal stages of the disease, these AR-independent GATA2 functions may become increasingly relevant.

Emerging strategies to therapeutically target GATA2 in prostate cancer include the GATA2 small molecule inhibitor K7174 which suppresses the expression of AR, AR splice variants, and AR target genes *via* a posttranscriptional mechanism of inhibition (48). K7174 also significantly reduces tumor growth in a murine xenograft model of CRPC (48). Dilazep, a vasodilator that is used to treat patients with hypertension, cardiovascular, and renovascular disorders, is a second pharmaceutical agent that blocks GATA2 DNA-binding, suppresses the expression of AR, and reduces tumor growth in a murine xenograft model of CRPC (50). These therapeutic options have not yet been tested clinically for men with prostate cancer, however.

### 3.2 ERG and the AR

Fusions of androgen-regulated genes with the ETS-related gene *ERG* are among the most common genomic alterations in prostate cancer. This commandeering, or hijacking, of promoter elements results in AR-regulated *ERG* overexpression (34). These gene fusions account for 46% of patients in the TCGA primary prostate cancer cohort (34). The most common gene fusion partner is *TMPRSS2*, although fusions with other androgen-regulated genes such as *SLC45A3* and *NDRG1* have also been reported (34).

In prostate cancer, the ERG protein also cooperates with the AR to influence disease progression. Primary prostate tumors that harbor a *TMPRSS2-ERG* gene fusion possess a unique set of active *cis*-regulatory elements (CREs) compared to tumors lacking this genomic rearrangement (51), suggesting a potential link between ERG and cistromic reprogramming. These CREs were preferentially bound by other key prostate cancer transcription factors, including FOXA1 and the AR. Interestingly, the reprogramming was observed in VCaP cells, which harbor the *TMPRSS2-ERG* gene fusion, but not in 22Rv1 cells which lack the gene fusion (51). This suggests that ERG expression modulates the cistromes of FOXA1 and the AR leading to altered transcriptional programs (51).

Notably, *TMPRSS2-ERG* fusions often occur in combination with *PTEN* loss and *TP53* mutations in both primary prostate cancer and CRPC (52). As such, several studies have sought to characterize the role of ERG within these specific genetic contexts. Using a genetically engineered mouse model that conditionally overexpresses the *ERG* transgene in the prostate luminal epithelium, Chen and colleagues showed that ERG expression amplifies AR chromatin binding without affecting its expression level (53). They also showed that while *Pten* loss itself suppresses AR signaling, ERG expression in the genetic background of *Pten* loss partially restores the AR transcriptome (53). They attribute this to a pioneer factor-like role of ERG, wherein pre-established enhancers are first bound by ERG, then by the AR (53). Subsequently, Blee and colleagues showed that, in the context of *Pten/Tp53* alterations, ERG is important for maintaining the luminal epithelial lineage in prostate cancer cells by repressing the expression of cell cycle genes and upregulating AR pathway genes (52). Correspondingly, overexpression of ERG re-sensitizes androgen-refractory, *PTEN* null, LNCaP (LNCaP-RF) xenografts to enzalutamide (52). Together these findings suggest that the partial restoration of the AR transcriptome by ERG expression in tumors with *PTEN* loss is responsible for the re-sensitization of these tumors to enzalutamide.

Recently, an organoid model derived from the *Pten-*null, ERG-overexpressing mice described by Chen and colleagues was used to investigate the effects of knocking out ERG in the context of *Pten*-null prostate cancer (54). *Pten-*null organoids exhibited loss of lumen structures and formed solid 3D spheres, reminiscent of prostatic intraepithelial neoplasia (PIN) (54). Combined PTEN loss and ERG overexpression led to the formation of finger-like protrusions that phenotypically resemble invasive adenocarcinoma (54). Interestingly, knocking out ERG in ERG-overexpressing, *Pten-*null organoids did not cause prostate cancer organoids to revert to their *Pten-*null organoid phenotype, but instead led to an almost normal, glandular-like morphology (54). Chromatin immunoprecipitation followed by next-generation sequencing (ChIP-seq) revealed that while ERG overexpression leads to a dramatic change in the AR cistrome in *Pten-*null organoids, the AR cistrome remains relatively unchanged following ERG knock-out (54). Nevertheless, ERG knock-out leads to significant reversal and dampening of the AR signature without downregulating its expression (54). This suggests that ERG overexpression leads to more permanent alterations to AR chromatin binding and results in partial restoration of AR signaling in the context of *PTEN* loss. Nonetheless, ERG also appears to regulate AR-dependent transcriptional targets through another mechanism. Using rapid immunoprecipitation mass spectrometry of endogenous protein (RIME), disrupted protein-protein interactions between the AR and transcriptional machinery, including RNA polymerase II and various elongation factors (54), were identified following ERG knock out. Indeed, ERG itself was found to significantly interact with the AR in ERG-overexpressing *Pten-*null organoids (54). Therefore, in addition to priming the AR cistrome for partial restoration of AR signaling in the context of *PTEN* loss, ERG may also function as a crucial AR cofactor that contributes to the transcriptional activation of AR target genes.

Thus, akin to FOXA1, HOXB13, and GATA2, ERG can also influence the AR cistrome. Of note, studies have also found a repressive role of ERG on the AR transcriptional program in VCaP cells (55, 56). Despite these findings, ERG did confer greater enzalutamide sensitivity in VCaP cells and in a VCaP-based *in vivo* model of bone tumor growth compared to ERG knock-down groups (55–57). These conflicting reports regarding the role that ERG plays on modifying the AR transcriptional program may be partially explained by the different genetic models used (58). For example, VCaP cells express PTEN, whereas the described mouse models were engineered on a *PTEN* loss background. Furthermore, ERG overexpression is itself insufficient for prostate cancer initiation and *ERG* gene fusions often co-occur with loss of function alterations to *PTEN/TP53* in both primary and metastatic prostate cancer. Therefore, it is possible that the genetic background of the tumor dictates the oncogenic consequences of ERG overexpression (52, 53). *PTEN* loss is additionally associated with anti-androgen insensitivity and the suppression of AR signaling, whereas VCaP cells are hormone-sensitive (52, 53, 55). Therefore, the tumor’s reliance on AR signaling may also dictate the role that ERG plays during disease progression. Nevertheless, there is convincing evidence that the oncogenic consequences of ERG overexpression are intimately tied to its ability to modulate the AR cistrome and AR signaling (**Figure 2**).

Despite initial promise in pre-clinical models, reports regarding the efficacy of androgen-targeted therapies in prostate cancer patients harboring *TMPRSS2-ERG* gene fusions have not been conclusive (59, 60). Since, the genetic background of the tumor may dictate how ERG influences AR activity, as described above, we propose that the results from patient cohorts may be obfuscated by mixed genetic backgrounds of patient tumor samples. Further characterization of the role that ERG plays in *PTEN-*intact prostate cancers and/or in hormone-sensitive prostate cancers may reveal their interwoven molecular dependencies and the molecular mechanisms driving disease progression potentially leading to new treatment stratification schemes.

### 3.3 c-MYC and the AR

The proto-oncogene c-MYC (MYC) is overexpressed in around 8% of primary prostate cancer cases in the TCGA cohort and up to 37% in metastatic disease (58, 61). Previous work has produced conflicting results regarding the influence of MYC on the AR transcriptome. Barfeld and colleagues described an antagonistic relationship between MYC and AR, and showed that androgen stimulation of hormone-sensitive prostate cancer cell lines led to a reduction in MYC at the transcript and protein levels (62). Reciprocally, MYC overexpression paired with androgen stimulation led to the downregulation of androgen-induced genes (62). However, results from a different study conducted by Bai and colleagues suggest a positive regulatory network between MYC and AR (63). Specifically, the AR and its isoforms were found to be positively correlated with MYC expression in primary prostate cancer and in CRPC (63). Furthermore, mCRPC samples with high AR expression were highly enriched for two hallmark MYC gene sets (63). Knocking down MYC in various human prostate cancer cell lines led to decreased expression of full-length AR and its splice variants, along with decreased expression of their target genes (63). Interestingly, both studies showed that there was no direct interaction between MYC and the AR, suggesting that MYC regulates AR target gene expression *via* an indirect mechanism (62, 63).

More recent evidence also suggests that MYC represses the AR transcriptional signature, and that the AR in turn suppresses MYC expression (64). In support of an antagonistic relationship between MYC and AR, the authors showed that genes upregulated following MYC-depletion *in vitro* were enriched for androgen-activated genes. On the other hand, MYC overexpression, *in vitro*, repressed global AR activity (64). Similarly, androgen deprivation and castration of tumor-bearing mice resulted in the upregulation of MYC in VCaP cells and VCaP xenografts, respectively. Androgen treatment repressed MYC expression in VCaP cells and VCaP xenografts of castrated mice (64). Additionally, the authors demonstrated that androgen-dependent downregulation of MYC occurred *via* a disrupted interaction between a prostate-specific super-enhancer and the *MYC* promoter in response to androgen stimulation (64). Altogether, the results provide further evidence of opposing functions for the AR and MYC in prostate cancer, as well as the role that chromatin remodeling plays in this dynamic.

In a concordant study by Qiu, Boufaied and colleagues, MYC overexpression in the murine prostate was shown to suppress AR transcriptional activity (65). In patients with CRPC where the AR was still expressed, AR activity was likewise negatively correlated with MYC expression. In line with previous reports, the suppression of the AR transcriptional program driven by MYC overexpression did not seem to be mediated by downregulation of the AR itself nor by disengagement of the AR from chromatin (62, 64, 65). Instead, evidence from pre-clinical models points to RNA polymerase II proximal pausing at AR-regulated genes as the potential mechanism mediating MYC-driven downregulation of the AR transcriptional signature (65). Altogether, the results from recent studies support a model of competition between the AR and MYC with transcriptional cofactor redistribution or sequestration as the primary mechanism driving MYC-dependent downregulation of the AR transcriptional signature.

Of clinical relevance, Qiu, Boufaied and colleagues revealed that a low AR and high MYC transcriptional signature in patient tumors was associated with shorter time to biochemical recurrence, increased risk of metastatic disease, and higher likelihood of developing resistance to ARSI treatment (65). Therefore, targeting MYC may be a viable therapeutic strategy in advanced prostate cancer patients, particularly those who have progressed to stages of the disease that are no longer sensitive to ARSI (**Figure 2**). Supportingly, pharmacological inhibition of MYC results in restored sensitivity to enzalutamide in cell lines and xenograft models that were previously enzalutamide-resistant (63, 66). Interestingly, this study by Holmes and colleagues also showed co-loss of FOXA1 and AR chromatin binding at promoter-distal regions in response to pharmacological MYC inhibition, suggesting that MYC inhibition may reprogram the AR cistrome by disturbing the cistrome of its cofactor and pioneer transcription factor FOXA1 (66). Thus, dissection of the interplay between MYC and AR has also uncovered potential avenues for therapeutic development.

While MYC is a proto-oncogene relevant in a wide variety of cancers, it has also been a challenging protein to target due to its nuclear localization, biochemistry, and its physiological relevance in normal tissues (67). Nevertheless, a suite of direct and indirect methods of targeting MYC are being developed and optimized, many of which were summarized by Whitfield and colleagues (67). Notably, inhibitors of the Bromodomain and Extra-Terminal motif (BET) proteins have remarkable downregulatory effects on MYC by disrupting super-enhancer regulatory networks that regulate its expression (68). However, BET inhibitors often have effects that extend well beyond their impact on MYC. For example, in *in vitro* models of prostate cancer, the BET inhibitor JQ1 interacts with FOXA1 and prevents it from repressing genes implicated in epithelial-to-mesenchymal transition, resulting in invasive phenotypes (69). Interestingly, JQ1 also has effects on HOXB13 and GATA2, blocking transcription at the *HOXB13* promoter, and inhibiting GATA2 DNA-binding respectively (70, 71). However, it should be noted that BET inhibitors encompass a large class of pharmaceutical agents that target BET proteins through heterogenous mechanisms. For example, JQ1 competitively binds to the bromodomain and displaces BET proteins from the chromatin while the small-molecule BET inhibitor dBET6 targets BET proteins for proteasomal degradation (72). The differences in biological mechanisms also accompany differences in biological outcomes. While JQ1 preferentially downregulates super enhancer-associated genes, including *MYC*, dBET6 does not exhibit this bias, instead resulting in a more global reduction of transcriptional elongation and different oncological outcomes *in vivo* compared to treatment with JQ1 (72). However, this was shown in models of T-cell lymphoblastoma, and studies on the impact of dBET6 treatment on MYC-driven prostate cancer are still lacking. Nevertheless altogether, these studies demonstrate that, although they can negatively impact MYC transcriptional activity, BET inhibitors do not exclusively target MYC and their mechanisms of action are multiple.

Small molecules that directly target MYC and its interaction with cofactor MAX, resulting in its proteasomal degradation, have also been developed and tested in a MYC-driven prostate cancer murine model, exhibiting remarkable anti-tumor effects (73). Another novel approach to target MYC in prostate cancer is through dietary modifications, as high-fat diets, more specifically high animal, or saturated fat diets, have been shown to enhance a MYC-driven transcriptional program (74). We propose that investigating the mechanistic link between high-fat diets and advanced prostate cancer would offer more options for designing effective treatment modalities for prostate cancer patients where MYC may play a key role in driving disease progression and mediating therapeutic resistance. Therefore, with a stronger understanding of the mechanisms mediating MYC-dependent disruption of AR signaling, we can better optimize and design tools for targeting its role in advanced disease.

## 4 Chromatin and epigenetic regulators alter the AR cistrome and contribute to disease progression

### 4.1 The chromatin modifying helicase CHD1

The chromatin modifying helicase CHD1 is a chromatin remodeler that has been implicated as a tumor suppressor in primary prostate cancer, as *CHD1* is recurrently deleted in primary prostate tumors (75, 76). Data from a human AR positive prostate cancer cell line, LNCaP, reveals that the set of AR and CHD1 interacting proteins overlap considerably, and CHD1-bound enhancer regions are highly concurrent with those bound by the AR (76). The CHD1 cistrome also overlaps with the cistromes of FOXA1, HOXB13, and ETV1. Each of these transcription factors has an established role in regulating AR-dependent transcription (76), which raises the possibility that CHD1 cooperates with other transcription factors to regulate the AR cistrome and AR signaling. Strikingly, deletion of *CHD1* in LNCaP cells leads to reprogramming of the AR cistrome and the resulting *CHD1-*null AR cistrome is enriched for HOXB13 motifs (76). Therefore, CHD1 may play a role in redistributing AR across the genome by regulating chromatin accessibility. The loss of *CHD1* in LNCaP and in patient tumors was also accompanied by the activation of what appears to be a *CHD1-*null subtype-specific AR transcriptional signature (76). Therefore, it appears that CHD1 acts as a tumor suppressor by preserving the integrity of the AR cistrome, whereas its loss leads to the reprogramming of the AR cistrome and an altered AR-associated transcriptome. However, further investigation of the tumor-suppressive functions of CHD1 in *CHD1-*intact prostate cancers is warranted. Seeing that the CHD1 cistrome overlaps with the cistromes of several transcription factors implicated in driving disease progression, it would be curious to understand the nature of the interactions between CHD1 and these transcription factors, and how CHD1 cooperates with these transcription factors to regulate the AR cistrome in the normal prostate and during prostate cancer onset.

Indeed, in the context of mCRPC and resistance to ARSI, CHD1 appears to take on a new role. Specifically, low *CHD1* mRNA levels were associated with shorter time to cancer progression among patients treated with ARSI including enzalutamide and apalutamide (77). *CHD1* loss *in vitro* and *in vivo* similarly accompanied enzalutamide-resistant prostate cancer growth (77). Mechanisms underlying resistance to ARSI are varied, and are reviewed more thoroughly elsewhere (13). However, in this case, *CHD1* loss did not appear to restore AR signaling in the context of therapeutic resistance to ARSI. Instead, profiling of the transcriptional and chromatin landscape of enzalutamide-resistant tumors by RNA-seq and by an assay for transposase-accessible chromatin paired with next-generation sequencing (ATAC-seq) revealed that *CHD1* loss led to widespread chromatin remodeling and the upregulation of divergent transcriptional outputs (77). These findings support a model of prostate cancer progression whereby the dysregulation of chromatin remodelers results in a plastic epigenomic state that permits master transcription factors to expand or adopt an altered cistrome (**Figure 2**). In the absence of ARSI, this allows the AR cistrome to evolve and continue driving AR-dependent prostate cancer growth, while in the presence of ARSI this enables other transcription factors to rise in dominance and drive cancer progression.

### 4.2 The chromatin remodelling complex SWI/SNF

The switch/sucrose-nonfermentable (SWI/SNF) complexes are chromatin remodelers that reposition nucleosomes using a catalytic subunit, either SMARCA4 or SMARCA2 (78). The SWI/SNF complex is a known cofactor of the AR and contributes to AR-dependent gene regulation in prostate cancer (79–82). Indeed, targeting the interaction between AR and SWI/SNF disrupts AR-dependent prostate cancer cellular proliferation (82, 83). In addition to being involved in AR-driven prostate adenocarcinoma, SWI/SNF is also involved in ARSI-refractory disease as well. The SMARCA4 (BRG1) subunit, as well as neuron-specific SWI/SNF subunits BAF53B, BAF45B, and CREST, are significantly overexpressed in NEPC compared to CRPC adenocarcinoma, thus positioning SWI/SNF as a regulator of lineage plasticity (84). Indeed, Cyrta and colleagues postulate that specialized forms of the SWI/SNF complex may be assembled in prostate cancer cells depending on their phenotype (84). Thus, it appears that SWI/SNF activity plays an important role in prostate cancer progression during both AR-dependent disease stages and ARSI-refractory disease stages (**Figure 2**).

Recently, it has been shown that targeting SWI/SNF activity blocks the enhancer-binding activity of transcription factors involved in prostate cancer progression, and results in a remarkable reduction in tumor growth in a mouse model of CRPC (85). Mechanistically, this is due to a rapid loss in chromatin accessibility that ensues proteolysis-targeting chimera (PROTAC)-mediated degradation of SMARCA4 and SMARCA2, particularly at enhancer regions (85). This is accompanied by the loss of DNA binding of several key transcription factors in prostate cancer progression, including the AR, FOXA1 and ERG (85). Accordingly, degradation of the SWI/SNF catalytic subunits led to the downregulation of AR, FOXA1, and ERG-regulated genes, as well as the downregulation of these transcription factors themselves, disrupting their enhancer circuitry in these cells (85). Multiple core components of the SWI/SNF complex interact with the AR, FOXA1, and ERG (85). Indeed, in addition to interacting with the AR to enable AR-dependent gene regulation, the SWI/SNF complex can be hijacked by ERG to target ERG-binding sites (86). Therefore, it seems that the SWI/SNF complex cooperates with these other factors to regulate enhancer-dependent transcriptional networks that are implicated in prostate cancer pathogenesis.

These findings position SWI/SNF as an exciting target in prostate cancer, where enhancer and cistromic reprogramming have been shown to play pivotal roles in disease progression. Indeed, the PROTAC degrader of SMARCA2 and SMARCA4, named AU-15330, also inhibited and, at times, reversed tumor growth in various xenograft models when administered either alone or in combination with enzalutamide. Importantly, no loss in body weight or histological evidence of toxicity in other organs was observed (85). AU-15330 is effective in inhibiting the growth of enzalutamide-resistant cell lines (85), however whether this is driven by the loss in chromatin accessibility at enhancers important for driving ARSI resistance warrants further investigation. Nevertheless, these results illustrate the key roles of non-coding regulatory elements in prostate cancer, and that targeting chromatin remodelers which regulate their accessibility is a viable therapeutic strategy.

### 4.3 The enhancer of zeste homolog 2 EZH2

The enhancer of zeste homolog 2 (EZH2) is a catalytic core subunit of the polycomb repressive complex 2 (PRC2) (39). Its canonical role is that of a transcriptional silencer, depositing Histone 3 Lysine 27 (H3K27) trimethyl (H3K27me3) marks across the genome, in cooperation with the Suppressor of Zeste 12 Protein Homolog (SUZ12) and Embryonic Ectoderm Development (EED) subunits of the PRC2 complex (39). EZH2 is notably upregulated in localized prostate cancer and particularly in metastatic prostate cancer compared to benign tissue (87). The oncogenic functions of EZH2 have been attributed to its ability to silence tumor suppressor genes (88). For example, Burkhart and colleagues demonstrated increased EZH2 expression and H3K27me3 levels in genetically engineered mouse models of prostate cancer relative to age-matched wild-type mice (89). Supporting the role of EZH2 in prostate cancer progression, a study by Labbé and colleagues found that prostate cancer patients with high EZH2 and DNA topoisomerase 2 alpha (TOP2A) expression had a distinct transcriptome that was coupled with shorter time to biochemical recurrence and progression to metastatic disease (90). In addition, EZH2-mediated trimethylation of endogenous retroviral DNA sequences leads to the inhibition of interferon-stimulated genes (ISGs) (91). Endogenous retroviral DNA sequences normally form double stranded RNAs (dsRNAs) that contribute to ISG activation. The loss of EZH2 catalytic function, by chemical inhibition, led to increased dsRNA levels, loss of H3K27me3 and concurrent gain of H3K27 acetylation (H3K27ac), a marker of transcriptional activation, at 302 genes containing endogenous retroviral sequences in *in vitro* and *in vivo* models of prostate cancer (91). This was accompanied by the upregulation of ISGs. Ultimately, Morel and colleagues demonstrate that ISG upregulation in response to EZH2 inhibition re-sensitizes tumors to checkpoint inhibitor therapy (91). Thus, EZH2 is an important player in prostate cancer initiation, progression, and therapeutic response, owing partially to its role in modulating the epigenomic landscape and ensuing tumor transcriptome *via* its catalytic methyltransferase function (**Figure 2**).

Notwithstanding its canonical role as a transcriptional silencer, EZH2 has several non-canonical functions in prostate cancer, which complicate therapeutic attempts to target it. For example, in addition to methylating histones, EZH2 also methylates FOXA1 and prevents its proteasomal degradation (39). Furthermore, EZH2 has an important role as a transcriptional co-activator in CRPC which is independent of its role as part of the PRC2 complex (92, 93). Specifically, EZH2 cooperates with the AR at a subset of promoter regions to activate transcription in an androgen-independent *in vitro* model of CRPC (93). Interestingly, this is independent of its involvement in the PRC2 complex, yet dependent on its intact methyltransferase domain (93). Of note, EZH2 also contributes to the expression of AR transcriptional signatures in models of both primary prostate cancer and CRPC *via* a different mechanism that is independent of both PRC2 and its methyltransferase activity (92). Therefore, in addition to its role as an epigenetic modifier that modulates prostate cancer cistromes, EZH2 also acts through additional direct and indirect mechanisms to regulate AR-driven prostate cancer progression (**Figure 2**).

Moreover, EZH2 contributes to the development of enzalutamide resistance by way of reprogramming AR binding activity. In enzalutamide-resistant cells, the AR was shown to interact with and co-occupy sites bound by a non-canonical polycomb complex consisting of EZH2 and SUZ12 but lacking EED (18). These co-occupied sites were often open, accessible chromatin regions, and converged on a set of transcriptional programs governing stem cell plasticity and neuronal differentiation (18). In this model, EZH2 was essential for establishing the lineage-infidelity state that resulted from sustained enzalutamide treatment (18). Interestingly, Dardenne and colleagues illustrated an alternative mechanism of treatment resistance and progression to NEPC, whereby EZH2 cooperates with N-Myc to downregulate AR target genes without disrupting AR expression (94). Specifically, Dardenne and colleagues showed that N-Myc directly binds with the AR at AR-bound enhancers, an interaction that is largely dependent on complex formation with EZH2 and SUZ12 (94). N-Myc overexpression led to an increase in EZH2 binding and H3K27 trimethylation at AR-binding sites (94). This is consistent with the canonical role of EZH2 as a histone methyltransferase. Altogether, these results suggest that the interactome of EZH2 is dynamic and responds to various therapeutic challenges in cooperation with the transcription factors that are available, to bring about divergent outcomes involving the AR.

These studies demonstrate the importance of chromatin and epigenetic remodelers in governing prostate cancer progression and attest to the diverse mechanisms of therapeutic resistance that are both AR-driven and AR-independent. In addition, they highlight the role that chromatin and epigenetic regulators play in influencing tumorigenic changes to the AR cistrome. It is tempting to hypothesize that targeting these chromatin and epigenetic regulators, which lie at the heart of therapeutic resistance, could re-sensitize tumors to ARSI. For example, treatment with EZH2 inhibitors in various *in vitro* and *in vivo* prostate cancer models of *PTEN* and *RB1* loss sensitized cells and tumors to enzalutamide (95). The roles of epigenetic regulators and remodelers are dynamic in prostate cancer progression, which highlights the need to evaluate genetic and epigenetic dependencies of prostate tumors in robust models that mimic the diversity of human prostate cancer patient backgrounds, especially in advanced, therapeutic-resistant stages such as NEPC.

## 5 Discussion

### 5.1 Tying it all together: The epigenome, the cistrome, and prostate cancer biology

Researchers have long sought to understand how ubiquitous genomes are translated into cell-type specific transcriptional networks. This is especially relevant in cancer contexts, where formerly faithful transcriptional networks often appear to be hijacked during tumorigenesis. In this review, we have provided examples of how the activities of transcription factors and epigenetic and chromatin remodelers are altered, and how these, in collaboration with the AR and its cistrome, ultimately affect prostate cancer progression. Together, these studies portray the chromatin and epigenetic landscapes in prostate tumors as dynamic and responsive to external stimuli.

It is abundantly clear that prostate cancer biology is distinct from normal prostate biology, and furthermore, that prostate cancer biology continues to evolve throughout disease progression and in response to therapeutic challenges. Pioneer transcription factors, including FOXA1, HOXB13, and GATA2, as well as the transcription factor ERG, have prominent roles to play in earlier, hormone-sensitive stages of the disease and in CRPC where AR signaling remains a mainstay of prostate tumor growth. MYC overexpression, which is observed in primary prostate tumors as well, has a profound role in the progression to a CRPC disease stage by counterbalancing the transcriptional activity of the AR and driving resistance to ARSI. The epigenetic and chromatin regulators CHD1, SWI/SNF, and EZH2 likewise play important roles in the evolution of the AR cistrome and prostate cancer biology. Notably, *CHD1* deletion is an early prostate cancer event, resulting in the loss of integrity of the AR cistrome. Interestingly, in the face of therapeutic challenge with ARSI, CHD1 loss seems to then allow alternative master transcription factors to dominate, leading to AR-independent mechanisms of tumor growth. Finally, SWI/SNF and EZH2 have relevant roles in both AR-dependent and AR-independent disease stages, partially owing to their ability to adopt different complex formations. In particular, the role of EZH2 in prostate cancer is multi-faceted, in that it can both promote and repress AR transcriptional activity during CRPC and in response to ARSI respectively, depending on the specific subunits it adopts and the cofactors that it interacts with. The relevance of each of these factors at various stages of prostate cancer disease progression is summarized in **Figure 2**.

It should be noted that there are other molecular mechanisms that contribute to the development and maintenance of the AR cistrome in prostate cancer as well. Although beyond the scope of this review, AR amplifications themselves have been shown to influence the AR cistrome in prostate cancer settings (96). In addition, mutations of the speckle type BTB/POZ protein SPOP, observed in about 10% of localized primary prostate cancers, have also been shown to drive the development of an oncogenic AR cistrome (34, 35, 97, 98). Interestingly, unlike the transcription factors and epigenetic regulators described in this review, SPOP is not a DNA-binding protein, and instead exerts its influence on the AR, at least in part, by regulating is ubiquitin-mediated degradation (99). Therefore, beyond creating or eradicating accessible sites on the chromatin for the AR to bind, and beyond directly recruiting the AR to particular genomic loci, other mechanisms can also contribute to an oncogenic AR cistrome.

Although the precise timelines of these molecular events may be challenging to dissect, recognizing the alterations to the epigenome and the AR cistrome that accompany disease progression can point to vulnerabilities in prostate cancer. A dynamic and plastic epigenome during disease progression hints that these alterations may be reversible. For example, emerging evidence suggests that global changes to chromatin underly resistance to ARSI and progression to AR-independent disease stages. One possible strategy to avert these lethal clinical outcomes would be to reprogram and restore the epigenome to an AR-dependent state in order to re-sensitize tumors to ARSI, as demonstrated by Ku and colleagues (**Figure 2**) (95). Recent technological advances have made it possible to systematically interrogate transcription factor cistromes through genome-wide CRISPR screens (100), and integrate epigenomic maps and gene expression data from multiple patients and cancer cell lines (14, 15, 21, 64, 101, 102). Paired with pharmacological advances that enable us to specifically target different factors in prostate cancer development, such as PROTACs, we are in a position where we can fine-tune the usage of these pharmaceutical agents to best suit the unique molecular profiles that we observe among patients and across different disease stages.

### 5.2 Concluding remarks

To summarize, while effective therapeutic options exist for most prostate cancer patients, resistance to ADT and ARSI remains an ongoing challenge among patients who progress to advanced stages of the disease. Reprogramming of the AR cistrome and subsequent alterations in AR-dependent gene expression underly prostate cancer progression. This plasticity is driven by key transcription factors, including FOXA1, HOXB13, GATA2, ERG, and MYC, as well as epigenomic and chromatin remodelers including SWI/SNF, CHD1, and EZH2. While these factors often subvert attempts to control the disease, they also present opportunities to exploit novel tumor dependencies. By uncovering mechanisms underlying epigenomic plasticity that challenge our standard treatments, we may uncover new ways to target this plasticity.

## Author contributions

Conceptualization, MS, L-KD, SB, and DPL. Writing—original draft preparation, MS. Writing — review and editing, MS, L-KD, SB, and DPL. Visualization, MS. Supervision, DPL. Funding acquisition, DPL. All authors contributed to the article and approved the submitted version.

## Funding

MS is a recipient of a Canadian Institute of Health Research (CIHR) Frederick Banting and Charles Best Canada Graduate Scholarship-Master’s. L-KD is a recipient of a Scholarship (Master) from the Fonds de Recherche du Québec – Santé (FRQS) and of a McGill Claude Gagnon Urology Research Studentship. SB is a Research Scholar – Junior 1 from the FRQS. DPL is a William Dawson Scholar of McGill University, a Lewis Katz – Young Investigator of the Prostate Cancer Foundation and is also a Research Scholar – Junior 2 from the FRQS. We acknowledge the support of the CIHR (project grant PJT-162246) to DPL.

## Acknowledgments

We thank Dr. Noriko Uetani for help with the design and drawing of figures.

## Conflict of interest

The authors declare that the research was conducted in the absence of any commercial or financial relationships that could be construed as a potential conflict of interest.

## Publisher’s note

All claims expressed in this article are solely those of the authors and do not necessarily represent those of their affiliated organizations, or those of the publisher, the editors and the reviewers. Any product that may be evaluated in this article, or claim that may be made by its manufacturer, is not guaranteed or endorsed by the publisher.
